# Supplementary material for: Risk Groups for Vaccine-Preventable Respiratory Infections in Children and Adults: An Overview of the Australian Environment
Source: Vaccines (Basel). 2025 Nov 30;13(12):1212. doi: 10.3390/vaccines13121212 (PMC12737586; doi:10.3390/vaccines13121212)
Supplement: Supplementary file 1 [file vaccines-13-01212-s001.zip › vaccines-3984140-Supplementary.pdf]

SUPPORTING INFORMATION

The search strategy for the narrative review is shown in the Table below. In total 319 articles from 2014 onwards were retrieved from the search, of which 244 articles were excluded based on title/abstract screening. Full text screening was performed on the remaining 75 articles. A total of 30 articles were retained and an additional 83 articles were identified through grey literature searches and hand searching of references lists of included papers. Whilst the search was restricted to identify articles reporting Australian data relevant publications of interest were included from other countries. Figure 1S shows the outcomes of the literature review for the different indications.

PubMed	Search string
**#1 Disease of interest**	(“Pneumococcal infection*”[tiab] OR “Streptococcus pneumonia”[tiab] OR “Pneumococcal pneumonia”[tiab] OR “Pneumococcic pneumonia”[tiab] OR “Pneumococcus pneumonia”[tiab] OR “Streptococcal pneumonia”[tiab] OR “Streptococcus pneumoniae pneumonia”[tiab] OR “Streptococcus pneumonia”[tiab] OR “Diplococcus pneumoniae”[tiab] OR “Micrococcus pneumoniae”[tiab] OR “Pneumococcus”[tiab] OR “Pneumococcus”[tiab] OR “Pneumococcus pneumonia”[tiab] OR “Pneumococc*”[tiab]) OR (“pneumonia”[tiab] OR “Community acquired pneumonia”[tiab]) OR (“Respiratory syncytial virus”[tiab] OR “Respiratory syncytial virus infection”[tiab] OR “Respiratory syncytial virus*”[tiab] OR “RSV”[tiab] OR “Human RSV”[tiab] OR “hRSV”[tiab] OR “Pneumoviridae”[tiab] OR “Seasonal respiratory syncytial virus*”[tiab] OR “Seasonal RSV”[tiab]) OR (“Influenza”[tiab] OR “Flu”[tiab] OR “Seasonal influenza”[tiab] OR “Seasonal flu”[tiab]) OR (“Influenza A virus”[tiab] OR “Influenza A”[tiab] OR “Influenza B virus”[tiab] OR “Influenza B”[tiab] OR “H1N1”[tiab] OR “A/H1N1”[tiab] OR “H3N2”[tiab] OR “A/H3N2”[tiab] OR “Yamagata”[tiab] OR “B/Yamagata”[tiab] OR “Victoria”[tiab] OR “B/Victoria”[tiab]) OR (“COVID-19”[tiab] OR “COVID-2019”[tiab] OR “Severe acute respiratory syndrome coronavirus 2”[tiab] OR “SARS-CoV-2”[tiab] OR “Coronavirus”[MeSH Terms] OR “Coronavirus”[tiab])
**#2 Risk populations and factors**	(“High risk*”[tiab] OR “High-risk population”[tiab] OR “High risk population*”[tiab] OR “Risk group*”[tiab]) OR (“Risk factor*”[tiab] OR “Predisposing factor*”[tiab] OR “Morbidity”[MeSH] OR “Morbidity”[tiab] OR “Comorbid*”[tiab] or “Multimorbid*” [tiab])
**#3 Countries of interest**	“Australia”[tiab] OR “Australian”[tiab]
**String combination**	#1 AND #2 AND #3
**Filters**	In the last 10 years, Humans, English<br><br>Clinical Study, Clinical Trial, Controlled Clinical Trial, Government Publication, Meta-Analysis, Observational Study, Randomized Controlled Trial, Systematic Review

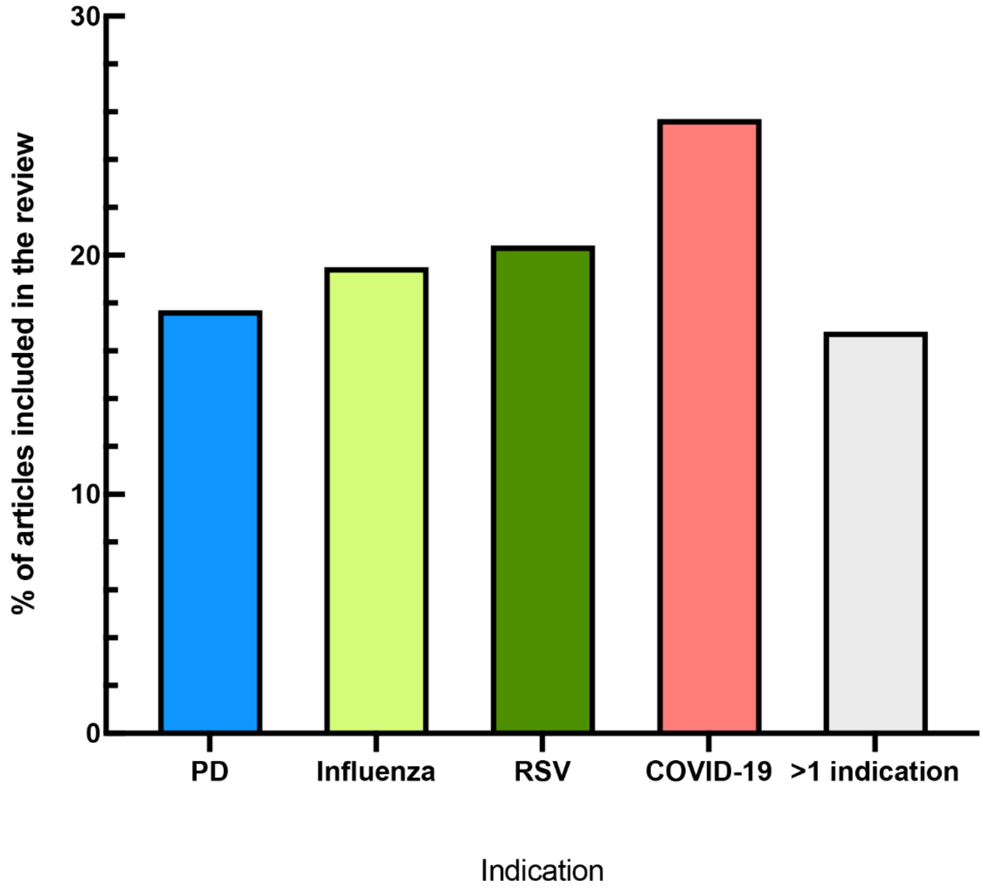

Figure S1 Literature search outcomes.

Table S1: Australian adult and child vaccine guidelines and recommendations for certain respiratory diseases[3]

Eligibility	Respiratory Vaccine						
	PD	NIP funded	Influenza	NIP funded	RSV	NIP funded	COVID-19
**Age-based recommendation**							
**Children**							
Age < 8 months					✓[a]		
Age 6 months to 5 years (annually)			✓	✓			
Age 6 months to 18 years							✓[b]
Age 2, 4, 12 months	✓	✓					
**Aboriginal and Torres Strait Islander children**							
Age 1, 4, 6, 12 months	✓	✓					
Age ≥ 5 years (annually)			✓	✓			
**Adults**							
Age ≥65 years			✓	✓			✓
Age ≥ 70 years	✓	✓					✓
Age ≥ 75 years					✓	✗	✓
**Aboriginal and Torres Strait Islander adults**							
All ages			✓	✓			✓
Age >50	✓	✓					
Age>60					✓	✗	

Eligibility	Respiratory Vaccine						
	PD	NIP funded	Influenza	NIP funded	RSV	NIP funded	COVID-19
**Clinical risk groups[c]**							
**Previous episode of disease**	✓	✓	✗	✗	✗	✗	✗
**Asplenia**							
Asplenia or splenic dysfunction	✗	✗	✓	✓	✓	✗	✓
Sickle cell disease or other haemoglobinopathies, Congenital or acquired asplenia (for example splenectomy) or hyposplenia	✓	✓	✗	✗	✗	✗	✓
**Chronic respiratory disease**							
Bronchiectasis, cystic fibrosis, suppurative lung disease	✓	✓	✓	✓	✓	✗	✓
Severe asthma, chronic obstructive pulmonary disease, chronic emphysema	✓	✗	✓	✓	✓	✗	✓
Interstitial and fibrotic lung disease	✓	✗	✗	✗	✗	✗	✗
**Chronic heart disease**							
Congenital heart disease, congestive heart failure, coronary artery disease	✓	✗	✓	✓	✓	✗	✓
**Chronic kidney disease**							
Relapsing or persistent nephrotic syndrome	✓	✓	✗	✗	✗	✗	✗
CKD stage 4 and 5	✓	✓	✓	✓	✓	✗	✓
**Chronic liver disease**							
Cirrhosis	✓	✗	✓	✓	✗	✗	✓
Autoimmune hepatitis, non-alcoholic fatty liver disease, alcoholic liver disease	✗	✗	✓	✓	✗	✗	✓
Chronic hepatis, biliary atresia	✓	✗	✗	✗	✗	✗	✗
**Diabetes mellitus**							
Type 1 diabetes, type 2 diabetes	✓	✗	✓	✓	✓	✗	✓
Any diabetes	✓	✗	✗	✗	✗	✗	✗
**Chronic Metabolic disorders**							
Amino acid disorders, carbohydrate disorders, cholesterol biosynthesis disorders, fatty acid oxidation defects, lactic acidosis, mitochondrial disorders, organic acid disorders, urea cycle disorders, vitamin/cofactor disorders, porphyria	✗	✗	✓	✓	✓	✗	✓

Eligibility	Respiratory Vaccine PD	NIP funded	Influenza	NIP funded	RSV	NIP funded	COVID-19
**Haematological disorder**							
Haemoglobinopathy	✗	✗	✓	✓	✗	✗	✓
**Immunosuppression**							
Congenital or acquired immune deficiency, including symptomatic IgG subclass or isolated IgA deficiency	✓	✓	✗	✗	✗	✗	✗
Haematological malignancies	✓	✓	✓	✓	✓	✗	✓
Malignancy	✗	✗	✓	✓	✓	✗	✓
solid organ transplant, haematopoietic stem cell transplant	✓	✓	✓	✓	✓	✗	✓
Immunosuppressive therapy, where sufficient immune reconstitution for vaccine response is expected; this includes those with underlying conditions requiring but not yet receiving immunosuppressive therapy	✓	✗	✓	✓	✓	✗	✓
CAR-T cell therapy	✗	✗	✓	✓	✓	✗	✓
HIV infection	✓	✓	✓	✓	✓	✗	✓
**Having cochlear implants**	✓	✓	✗	✗	✗	✗	✗
**Cerebrospinal fluid leaks**	✓	✓	✗	✗	✗	✗	✗
**Chronic neurological disease**							
Hereditary and degenerative central nervous system diseases, seizure disorders, spinal cord injuries, neuromuscular disorders, other conditions that increase the risk of severe outcomes from respiratory infection	✗	✗	✓	✓	✓	✗	✓
**Trisomy 21**	✓	✓	✓	✗	✗	✗	✗
**Obesity (BMI ≥30 kg/m²)**	✗	✗	✓	✗	✓	✗	✓
**Pregnancy (at all stages)**	✗	✗	✓	✓	✓[d]	✓[e]	✓
**Smoking**	✓	✗	✗	✗	✗	✗	✗
**Harmful use of alcohol**	✓	✗	✓	✗	✗	✗	✗
**Occupational risk (adults)**							
Health care workers, carers of people in high-risk groups	✗	✗	✓	N/A	✗	✗	✗
Staff, volunteers, visitors to aged care facilities	✗	✗	✓	N/A	✗	✗	✗
People working in early childhood education and care	✗	✗	✓	N/A	✗	✗	✗

Eligibility	Respiratory Vaccine						
	PD	NIP funded	Influenza	NIP funded	RSV	NIP funded	COVID-19
Staff or volunteers caring for homeless people	✘	✘	✓	N/A	✘	✘	✘
People providing essential services	✘	✘	✓	N/A	✘	✘	✘
Individuals working in poultry industry	✘	✘	✓	N/A	✘	✘	✘
**Other risk groups**							
Residents if care homes or other long-term facilities	✘	✘	✓	N/A	✘	✘	
Household contacts of people in high-risk groups	✘	✘	✓	N/A	✘	✘	
Homeless people	✘	✘	✓	N/A	✘	✘	
At-risk individuals at clinician's discretion	✘	✘	✓	N/A	✘	✘	✓

[a] Monoclonal antibody
[b] Only children with medical conditions that may increase their risk of severe disease or death from COVID-19 are recommended for vaccination.
[c] These risk groups also apply to children for PD, influenza and COVID-19 vaccination. Specific risk groups for children for RSV vaccination is outlined in Table S2 in Supporting Information.
[d] Vaccination at 28 to 36 weeks gestation.
[e] NIP funding to start in 2025.
Abbreviations: BMI, body mass index; CKD, chronic kidney disease; COVID-19, coronavirus disease 2019; HIV, human immunodeficiency virus; N/A: not applicable; PD, pneumococcal disease; RSV, respiratory syncytial virus; TIA, transient ischaemic attack.

Table S2: Additional recommendations for RSV vaccines for infants with risk conditions for severe RSV disease[3]

Eligibility[a]
Pre-term birth (< 32 week gestational age)
Hemodynamically significant congenital heart disease
Significant immunosuppression (such as solid organ transplant, haematopoietic stem cell transplant, or primary immune deficiency such as severe combined immunodeficiency)
chronic lung disease that requires ongoing oxygen or respiratory support
neurological conditions that impair respiratory function
cystic fibrosis with severe lung disease or weight for length <10th percentile
trisomy 21 or another genetic condition that increases the risk of RSV
**OR Infant has suboptimal RSV antibodies because they were born to a mother who**
received RSV vaccine in pregnancy at a time of severe immunosuppression
had a treatment associated with loss of maternally derived antibodies (such as cardiopulmonary bypass or extracorporeal membrane oxygenation)

[a] vaccine is recommended regardless of maternal vaccination. Treating doctor to confirm a clinical benefit
